# Schizophrenia and disordered sensorimotor control: challenges, mechanisms and opportunities

**DOI:** 10.1093/brain/awag064

**Published:** 2026-03-07

**Authors:** Albert Joseph, Rick A Adams, Fiona Gaughran, Oliver D Howes, Davide Martino, Francesca Morgante, Mark J Edwards

**Affiliations:** Institute of Psychiatry, Psychology, and Neuroscience, King’s College London, London SE5 8AF, UK; Department of Neurology, Royal London Hospital, London E1 1FR, UK; Centre for Medical Image Computing, Department of Computer Science, University College London, London WC1V 6LG, UK; Institute of Cognitive Neuroscience, University College London, London WC1N 3AZ, UK; Institute of Psychiatry, Psychology, and Neuroscience, King’s College London, London SE5 8AF, UK; Department of Psychiatry, South London and Maudsley NHS Foundation Trust, London SE5 8AZ, UK; National Institute for Health Research, Maudsley Biomedical Research Centre, South London and Maudsley National Health Service Foundation Trust, London SE5 8AF, UK; Institute of Psychiatry, Psychology, and Neuroscience, King’s College London, London SE5 8AF, UK; Department of Psychiatry, South London and Maudsley NHS Foundation Trust, London SE5 8AZ, UK; National Institute for Health Research, Maudsley Biomedical Research Centre, South London and Maudsley National Health Service Foundation Trust, London SE5 8AF, UK; MRC London Institute of Medical Sciences, Faculty of Medicine, Imperial College London, Hammersmith Hospital Campus, London W12 0HS, UK; Department of Clinical Neurosciences and Hotchkiss Brain Unit, University of Calgary, Calgary T2N 1N4, Canada; Neurosciences and Cell Biology Institute, Neuromodulation and Motor Control Section, City St George’s University of London, London EC1V 0HB, UK; Institute of Psychiatry, Psychology, and Neuroscience, King’s College London, London SE5 8AF, UK

**Keywords:** schizophrenia, psychosis, neuropsychiatry, movement disorders, tardive dyskinesia, parkinsonism

## Abstract

Schizophrenia is a common and often disabling neuropsychiatric condition. Whilst sensorimotor abnormalities such as dyskinesia, parkinsonism and motor incoordination are prevalent in schizophrenia, they are often attributed to medication side effects or classified as neurological soft signs or catatonic phenomena. Here, we outline the prevalence, characteristics and challenges in accurate phenotyping of sensorimotor disturbances in schizophrenia, including amongst medication-naïve individuals, demonstrating that sensorimotor dysfunction may be an integral manifestation of the disease process. We then review how current understanding regarding the pathogenesis of schizophrenia supports this possibility and consider how better characterization of sensorimotor dysfunction may improve management and the development of novel treatments for schizophrenia, playing particular attention to the role of instrumental sensorimotor assessment.

## Teaser

Whilst schizophrenia is known to affect thoughts and feelings, many patients also experience problems with movement, which are often attributed to medication. Joseph *et al.* explore evidence suggesting that sensorimotor abnormalities may be a core part of the disease and discuss how this new understanding could improve treatment.

## Introduction

The symptoms of schizophrenia emerge across neuropsychiatric domains.^1^ Positive symptoms implicate disruption in systems necessary for belief formulation and veridical perception alongside coherent organization of thought, speech and behaviour.^1^ Similarly, negative symptoms indicate impairment in processes underlying volition, social engagement and cognition.^1^ However, whilst motor abnormalities such as dyskinesia and parkinsonism are recognized to be highly prevalent in schizophrenia,^2,3^ they tend to not be regarded as part of the primary disease process. Instead, they are typically attributed to the side effects of medications used to treat schizophrenia^3,4^ despite a range of studies reporting their apparent occurrence in medication-naïve patients,^2,5-10^ though whether terms such as dyskinesia and parkinsonism should apply within this context is unclear.

When not ascribed to the effects of medication, motor deficits are categorized into rather confusing, non-specific entities. For example, a range of sensorimotor phenomena have been grouped together as neurological soft signs (NSSs),^11,12^ traditionally a term used to describe ‘minor’ abnormalities^13^ found on neurological examination that are either difficult to detect,^14^ or cannot be attributed to localized abnormalities within a specific brain region or tract.^13^ In practice, however, these signs are often not as ‘soft’ as the name implies,^13,15^ and can be localized to specific structures or networks within the sensorimotor system.^2,13,16^

Here, we review current knowledge on the sensorimotor deficits associated with schizophrenia, including those occurring in medication-naïve patients, relating to medication side effects and in the context of catatonia. We then provide an account for how these sensorimotor phenomena might develop, grounded in both the broader neuroscience of sensorimotor control and current hypotheses regarding the neurobiology of schizophrenia. We also explore how improved understanding of sensorimotor abnormalities in schizophrenia and related conditions might enable improved diagnostic and prognostic precision alongside enhanced treatment.

## Movement disorders in schizophrenia

### General overview

Schizophrenia affects ∼1% of the population,^1^ often leading to dramatic reductions in quality of life alongside social and occupational function.^1^ Life expectancy of people with schizophrenia is estimated to be 10–15 years lower than that of the general population, with a 5%–10% lifetime risk of suicide.^1^ Diagnosis relies on recognition of characteristic symptoms which must be persistent and cause functional impairment.^1^ Schizophrenia demonstrates a high heritability (up to 80%).^17^ Presentation with ‘first-episode psychosis’ (FEP) is often preceded by a prodromal ‘clinical high-risk’ (CHR) period, associated with functional decline and quasi-psychotic symptoms.^18^ The mainstay of pharmacological treatment is with dopamine-2 receptor (D2R) antagonists.^1,3,19^ These medications are highly effective in ameliorating and preventing relapse of positive symptoms but neutral in regard to their effect on negative symptoms.^20^

Following the widespread use of these medications from the 1950s onwards, it became apparent that they could have adverse motor side effects.^21-25^ Whilst some of these could be improved through treatment with anticholinergics, others did not respond or were worsened.^19,26^ The earliest D2R antagonists in use, the first-generation agents (FGAs), gradually came to be replaced by second-generation agents (SGAs), which were thought to have an improved side effect profile in respect to motor disturbance.^19^ However, movement disorders remained prevalent in patients with schizophrenia, even following their introduction.^3^

In schizophrenia, the label ‘spontaneous movement disorder’ has been frequently applied in cases where motor symptoms occur in D2R antagonist-naïve patients and these are then often further subdivided by phenotype—for instance ‘spontaneous dyskinesia’ or ‘spontaneous parkinsonism’.^5,6,8-10,27-29^ Importantly however, consensus guidelines around appropriate nomenclature in these cases are lacking and warrant further attention. ‘Extrapyramidal side effects’ is an umbrella term for all motor symptoms which are assumed to be related to medication and can present either acutely or as tardive syndromes. The latter is derived from the Latin for late, *tardus*, and describes the delayed side effects of D2R antagonists, which typically persist even following withdrawal of the medication.^30^ In adults, tardive syndromes are defined as occurring at least 3 months following treatment initiation, or after at least 1 month for those above the age of 60.^30^ Whilst tardive syndromes are further distinguished based on phenotypic description^4^ the concept of ‘tardive parkinsonism’ is controversial^3^ and often terms such as ‘drug associated’ or ‘induced parkinsonism’ are used as alternatives.

It is worth noting that many of the challenges we discuss later in relation to phenotyping and classification of motor disturbances are not unique to schizophrenia, as patients with many neuropsychiatric conditions, including Parkinson’s disease or Huntington’s disease, can exhibit multiple sensorimotor signs and symptoms, often with overlapping phenomenology. There are also general inherent limitations to the process of clinical phenotyping, in part stemming from the imprecision of available descriptive terms^31^ and high levels of inter- and intra-observer variability.^32-34^ This is an important point as in practice, the original phenotyping process will determine the clinical approach to patients^31^ and recruitment of participants to research studies. Novel multimodal study designs incorporating kinematic analysis and machine learning are currently being implemented in the broader movement disorders field to improve classification of motor disturbances and correlation with underlying pathophysiologic mechanisms.^35^ It is hoped that similar techniques, in combination with more careful clinical phenotyping, offer the scope to overcome some of the challenges outlined later in the context of schizophrenia.

### Dyskinesia

In movement disorder phenomenology, the term dyskinesia generally refers to any abnormal involuntary movement. However, in the context of schizophrenia, it describes a more specific set of repetitive, rhythmic movements, typically affecting the lower face and tongue but potentially extending to other body parts such as the periorbital areas, limbs, trunk, diaphragm and pelvis.^3,4^ While some researchers regard these movements as stereotypies,^36^ others argue against this categorization given they are neither fully distractible nor predictable,^3^ and therefore do not align with proposed diagnostic criteria.^37,38^ Additionally, whilst they are defined in the Diagnostic and Statistical Manual Fifth Edition^37^ (DSM-5) as ‘choreiform’ or ‘athetoid’ this categorization is also lacking, as movements are at least partially predictable (stereotyped)^3^ and do not typically flow between body regions.^39^ So, for the purposes of this review, the term dyskinesia is maintained, acknowledging the need for more accurate phenotyping of this condition.

At present, these movement abnormalities remain almost universally regarded as delayed side effects of D2R antagonists, referred to as tardive dyskinesia (TD).^3,4,36^ A meta-analysis of 41 studies published since 2000 estimated a global mean prevalence of presumed TD amongst patients exposed to D2R antagonists to be 25.3%, where 77.1% of individuals included in the study were determined to have schizophrenia-spectrum disorders.^40^ It is more common in the elderly^30,41^ and prevalence in one study reached 93% in patients with schizophrenia above the age of 75.^42^ It is also associated with prolonged use of D2R antagonists^3^ so that, after more than 10 years of exposure, prevalence is estimated to reach at least 50%.^30,43^ Additional risk factors include female sex, African ethnicity and history of brain injury or dementia.^30^ Following introduction of SGAs, it was thought that rates of TD would fall. Two initial large randomized controlled trials, Clinical Antipsychotic Trials of Intervention Effectiveness (CATIE)^44^ and Cost Utility of the Latest Antipsychotic Drugs in Schizophrenia Study (CUtLASS)^45^ comparing treatment outcomes with SGAs against FGAs, were initially thought not to support this, although the methodological validity of these trials has since been challenged.^3^ More recently, a meta-analysis has suggested a significantly lower rate of TD amongst people treated with SGAs; 20.7% versus 30% with FGAs.^40^

Although dyskinesia in schizophrenia is commonly attributed to antipsychotic treatment, there is evidence that abnormal involuntary movements may also occur in the absence of D2R antagonist exposure. However, the extent to which these movements, often referred to as ‘spontaneous dyskinesias’,^5,8,10,27,28^ represent an overlapping or distinct phenotype from what would be regarded as dyskinesia in medicated patients remains unclear and is a vital area for further study.

Such movements were described in the pre-neuroleptic era. For instance, in 1919, Kraepelin^46^ (p83) noted schizophrenic patients exhibiting ‘smacking and clicking with the tongue, sudden sighing … and clearing the throat … in the lip muscles, fine lightning-like rhythmical twitching, which in no way bear the stamp of voluntary movements’. Historical case records (1845–90) from the Ticehurst Asylum in Sussex, UK, also suggest a 28% prevalence of abnormal orofacial movements amongst patients found retrospectively to meet diagnostic criteria for schizophrenia, though there is a strong possibility that many patients had alternate diagnoses.^47^ Similarly, when case notes from a North American psychiatric cohort (1950–75) were reviewed retrospectively, 23.4% of medication-naïve patients with presumed schizophrenia were deemed to have abnormal involuntary movements, described by the authors as dyskinesia, compared with 7.3% in those with other psychiatric diagnoses, with most striking differences evident in rates of abnormal movements in the orofacial region (14.9% versus 1.7%), although periorbital regions and upper extremities were also more affected and gait disturbance also more common.^9^

Owing to widespread and rapid uptake of D2R antagonists from the 1950s, it became increasingly difficult to identify medication-naïve subjects for comparison to establish the extent to which dyskinesia occurred spontaneously. Despite the rapid acceptance that dyskinesia was a medication-driven process, data supporting this were rather limited, and the existence of spontaneous motor disturbance was often acknowledged in earlier literature published shortly after the advent of antipsychotics.^22,23,25^ Frequently cited evidence stemmed from a large cross-sectional study of 3775 patients, which identified a relationship between onset and severity of what were described as ‘extrapyramidal symptoms’ and both timing of initiation and potency of the D2R antagonist used as treatment.^21^ However, there were no non-medicated patients included for comparison in this study and most patients developed either akathisia (21.2%) or parkinsonism (15.4%) rather than dyskinesia (2.3%).^21^ Where medication-naïve patients were studied, groups were often poorly characterized,^22,23,25^ and movement abnormalities were sometimes disregarded due to suspicions that many might have suffered from neurological conditions such as epilepsy or Huntington’s disease (which is certainly a possibility). Reasons why certain individuals did not receive medication were also rarely stated,^22-25^ again limiting comparisons. Furthermore, at least to our knowledge, in none of the studies where movement abnormalities were attributed to prolonged medication exposure were comparisons made to non-medicated individuals over extended time periods. This is a crucial point: given that the prevalence of assumed TD diagnosis increases with age and duration of exposure,^3,30^ it is not inconceivable that in some cases D2R antagonists accelerate onset of disease-related dyskinesia rather than causing it directly.

Later investigators sought circumstances where it remained possible to contrast prevalence between presumed dyskinesia D2R antagonist exposed and naïve patients with schizophrenia and related conditions. For instance, Owens *et al*.^7^ assessed 47 patients with chronic schizophrenia, treated over several decades by a psychiatric team who had rejected use of D2R antagonists in favour of family therapy. The rates and severity of abnormal involuntary movements were strikingly similar in the neuroleptic-naïve patients (35.4% abnormal tongue, 28.1% abnormal lip movements) and a group of 364 similar (though slightly younger) medication-exposed patients treated in the same hospital.^7^ Abnormal movements demonstrated roughly similar distributions with respect to body part in both groups, most commonly affecting the lower face and mouth.^7^ However, on one of the two motor rating scales used,^48,49^ the Rockland,^48^ neuroleptic-naïve patients were significantly more likely to exhibit ‘head nodding’, and in subgroup analysis of patients with the most severe motor disturbance, exposed patients were significantly more likely to experience severe lingual choreoathetosis,^7^ again raising the question of whether dyskinetic-like movements seen in neuroleptic-naïve and -exposed individuals reflect the same entity. Unfortunately, information regarding the patients’ dental status was also not provided,^7^ which is relevant because edentulous (lacking teeth) patients can also develop oral dyskinesia.^49^

Other examples where medication-naïve patients have been assessed for motor disturbance include during FEP prior to medical treatment and in developing world contexts where some patients do not have access to antipsychotic treatments. Upon recent meta-analysis of 23 identified studies in D2R antagonist-naïve populations with psychosis involving 2340 patients, the overall random-effects pooled prevalence of reported spontaneous dyskinesia was 7% [95% confidence interval (CI): 3–11].^5^ There was, however, substantial sample heterogeneity identified (I^2^ = 94%, *P* < 0.01), which was only partially explained by differences between groups with FEP versus chronic psychosis, where pooled prevalence was 3% and 17%, respectively.^5^ Significant associations were identified between dyskinesia and both age (*P* < 0.05) and duration of untreated psychosis (*P* < 0.05).^5^ Whilst heterogeneity was not explained by variability in rating tools used for determining the presence of movement disorders, there remains a subjective element in determining what an individual observer may regard as dyskinesia or otherwise. Furthermore, of all the studies included, none met the ‘low-risk’ criteria of the meta-analytical protocol, suggesting significant methodological limitations, and definitions for psychosis were also broad, including affective psychosis.^5^ It is also difficult to the establish the extent to which use of both prescribed and illicit psychoactive substances could have contributed to the development of apparent dyskinesia within these populations.

Movements reported as spontaneous dyskinesia have also been observed at increased rates in adolescents during the CHR period, even when excluding concomitant use of recreational drugs,^28,50-55^ and has been noted to have a preponderance of jaw and lip movements,^50,52^ though one study identified higher rates of abnormal involuntary movements in tongue, trunk and lower limbs.^55^ In a separate meta-analysis, dyskinesia has also been found more prevalent in non-affected first-degree relatives of people with schizophrenia^6^ and, in a later study, dyskinetic movements correlated with positive schizotypal personality traits in their healthy siblings,^56^ suggesting a possible genetic contribution.

It is important to note that, even in individuals without schizophrenia, prolonged use of dopamine receptor antagonists such as the anti-emetic metoclopramide can produce a syndrome which is essentially indistinguishable from TD,^57^ supporting the importance of dopamine receptor blockage in the development of this disorder. Although metoclopramide-associated TD appears to be a relatively rare adverse effect,^58,59^ the extent and duration of D2R antagonism in patients receiving metoclopramide may be expected to be less than for patients with schizophrenia treated with antipsychotics. Furthermore, whilst recent epidemiological studies support the notion that antipsychotic-exposed patients with schizophrenia are more likely to develop TD when compared with patients with other conditions, they also demonstrate that the phenomenon is not unique to schizophrenia. For instance, in a prospective multicentre study based in North America, where 739 patients were enrolled with 3 months or more exposure to antipsychotic treatment and at least one psychiatric diagnosis, whilst participants with possible TD were significantly more likely to have diagnosis of schizophrenia or schizoaffective disorder (174/285, 61%), high rates were also evident in patients with mood or other psychiatric disorders (112/513, 21.8%).^60^ Importantly, patients with possible TD were also more likely to have had more prolonged antipsychotic exposure.^60^ Additionally, in a retrospective observational health registry study of 164 417 antipsychotic-exposed individuals, TD rates in patients with schizophrenia or related conditions were 4.51% (601/13 308) and 1.43% (277/19 359), respectively, compared with 0.87% (661/75 672) in those with mood disorders and 0.8% (1314/164 417) in all exposed patients.^61^ Notably, since these studies did not control for treatment-related factors such as duration, total dosage and frequency of interruptions to antipsychotic treatment alongside relative exposure to FGAs, it is unclear whether differences between groups are due to the underlying susceptibility, pattern of medication exposure or other confounding factors. It is certainly not unreasonable to assume that patients with schizophrenia or related conditions would receive more prolonged treatment courses at higher doses.

Further work is needed to establish the prevalence and distribution of dyskinesia in antipsychotic-naïve patients with schizophrenia, preferably using kinematic analysis to more accurately characterize hyperkinetic motor disturbances and compare to those observed in medication-exposed cohorts. Whilst there appears to be relatively strong data to support the notion that spontaneous dyskinetic movements do occur within these cohorts, the extent to which the underlying disease predisposes to what would typically be described as TD remains an important but unanswered question.

### Dystonia, stereotypies and other hyperkinetic movement disorders

Dystonia is characterized by sustained or intermittent muscle contractions causing abnormal, often repetitive, movements and postures that are typically patterned, twisting and sometimes tremulous.^62^ It is particularly prevalent amongst young men with schizophrenia, often occurring acutely, following initiation of D2R blocking treatment, presenting after 12 h to 5 days in 90% of cases,^41^ or as a tardive phenomenon, typically observed alongside other tardive syndromes.^41^ Axial structures are most frequently affected, including the face (blepharospasm, oculogyric crisis), neck (retro- or laterocollis) and trunk (hyper-extension with the appearances of opisthotonos or tonic lateroflexion leading to ‘Pisa syndrome’).^3^ In a meta-analysis of studies of antipsychotic-naïve patients with FEP and chronic psychosis,^5^ dystonia prevalence was highly heterogenous with rates of 15% and 16% in two studies^63,64^ whilst three further trials did not report any cases.^65^ Further work would be needed to establish the relationship between prevalence of dystonia and dyskinesia in medication-naïve patients, given hyperkinetic motor disturbance may be expected to overlap. Estimated prevalence rates vary considerably amongst medicated cohorts, ranging from 2.9% where only moderate to severe cases were considered, to 20% when very mild presentations were also considered.^66^

Stereotypy can be defined as ‘a non-goal-directed movement pattern that is repeated continuously for a period of time in the same form and on multiple occasions, and which is typically distractible’.^38^ Stereotypies are typically reported to be tardive phenomena^3,36^ or catatonic behaviours^67^ and again further work is required to defined more precisely what the term should mean within varying contexts. In untreated patients with FEP, stereotypies have been shown to be associated with both positive^68^ and negative symptoms,^69^ especially disorganization,^69^ alongside earlier disease onset^70^ and poor pre-morbid adjustment,^69^ which is notable given that stereotypies are frequently observed in neurodevelopmental conditions such as autism. Stereotypies were also found in 11 (5.5%) in a cohort of 200 medication-naïve patients with FEP.^64^

A range of other hyperkinetic motor abnormalities are seen in schizophrenia and frequently attributed to medication side effects,^3,4^ including non-parkinsonian tremor, myoclonus, chorea and tics.^3,4^ A ‘withdrawal emergent’ dyskinesia can also occur following cessation of D2R antagonists.^3,4^ The extent to which hyperkinetic motor disturbances, including dystonia and stereotypies, occur in unmedicated cohorts is an important area for further study.

### Akathisia

Akathisia is characterized by a feeling of inner restlessness combined with an inability to remain still.^3^ Rocking from foot to foot, walking in place, crossing/uncrossing legs or body rocking are used as objective indicators.^3^ It might be best described as sensorimotor disorder, given that the sensory component often appears to drive the motor features,^71^ though in ‘pseudo-akathisia’ the appearance of restlessness occurs without the subjective component.^3^ It is often attributed to D2R antagonist exposure, in which circumstances it may occur as an acute, dose-related adverse event, or following rapid medication withdrawal or as a tardive phenomenon.^71^ Across these contexts, clinical features are similar, with prevalence estimates varying widely in medicated patients ranging from 8% to 76%.^71^ It is not particularly well characterized as a spontaneous phenomenon in schizophrenia. In a meta-analysis of eight studies common and random-effects prevalence was 4% (95% CI: 3–6), again with substantial heterogeneity between studied populations.^5^

### Parkinsonism and hypokinetic motor disturbance

Parkinsonism is a hypokinetic motor disorder defined by the presence of bradykinesia in combination with rigidity, rest tremor or both, and often associated with postural instability.^72^ PET ligand studies of medicated patients with schizophrenia have shown that, when occupancy of striatal postsynaptic dopamine D2Rs exceeds 80%, parkinsonian symptoms may be inevitable.^1,73^ Unsurprisingly therefore, 20%–35% of patients exposed to dopamine D2R antagonists develop parkinsonism.^74^ However, despite the widespread association with D2R antagonism, parkinsonism or at least something akin to it, might also occur spontaneously in schizophrenia. Kraepelin,^46^ in 1919, observed patients for whom ‘the face appears vacant, immobile, like a mask’ and ‘simple movements are stiff, slow, forced’. Furthermore, numerous authors writing in the pre-neuroleptic era comment on slowing of movement in patients with schizophrenia.^75^ It is also notable that in 30%–40% of cases of presumed drug-induced parkinsonism, motor symptoms will remain or worsen following withdrawal of D2R antagonists.^76-78^ In a recent meta-analysis of three studies, a strong association between apparent parkinsonism and schizophrenia was identified [odds ratio (OR) 5.32] when medication-naïve patients were compared with healthy controls.^6^ There is a small but statistically significant increase in parkinsonism amongst non-affected relatives of patients with schizophrenia and, similar to spontaneous dyskinesia, parkinsonism was positively correlated with age and duration of untreated disease.^6^ Parkinsonism also appeared to be more common than dyskinesia within the same meta-analysis; in FEP and chronic psychosis prevalences of 14% (95% CI: 10–19) and 19% (95% CI: 12–28) were observed, respectively, with an overall pooled prevalence of 15% (95% CI: 12–20) with significant heterogeneity (I^2^ = 81%, *P* < 0.01), across 20 studies involving 1707 participants.^5^ Bradykinesia, measured through handwriting kinematics, is also increased amongst unmedicated CHR adolescents.^29^

It is important to consider the significant potential for diagnostic confusion between ‘true’ bradykinesia, and other hypokinetic motor disturbances in schizophrenia,^79^ including catatonia, negative symptoms and psychomotor slowing, in which impaired cognition results in slowness of movement.^75^ Indeed, five of six studies exploring the relationship between parkinsonism and negative symptoms in FEP demonstrated a positive association.^27^ Signs of bradykinesia, psychomotor slowing and catatonia also showed close cross-correlation within a subset of patients with chronic schizophrenia who also experienced greater severity of negative symptoms.^80^ The observed relationship between spontaneous parkinsonism and poor treatment response might also be due to its association with negative symptomatology.^27^ Again, further robust work is required to identify the prevalence of parkinsonism in those diagnosed with schizophrenia, not exposed to D2R antagonists.

### Catatonia

**Figure 1:**
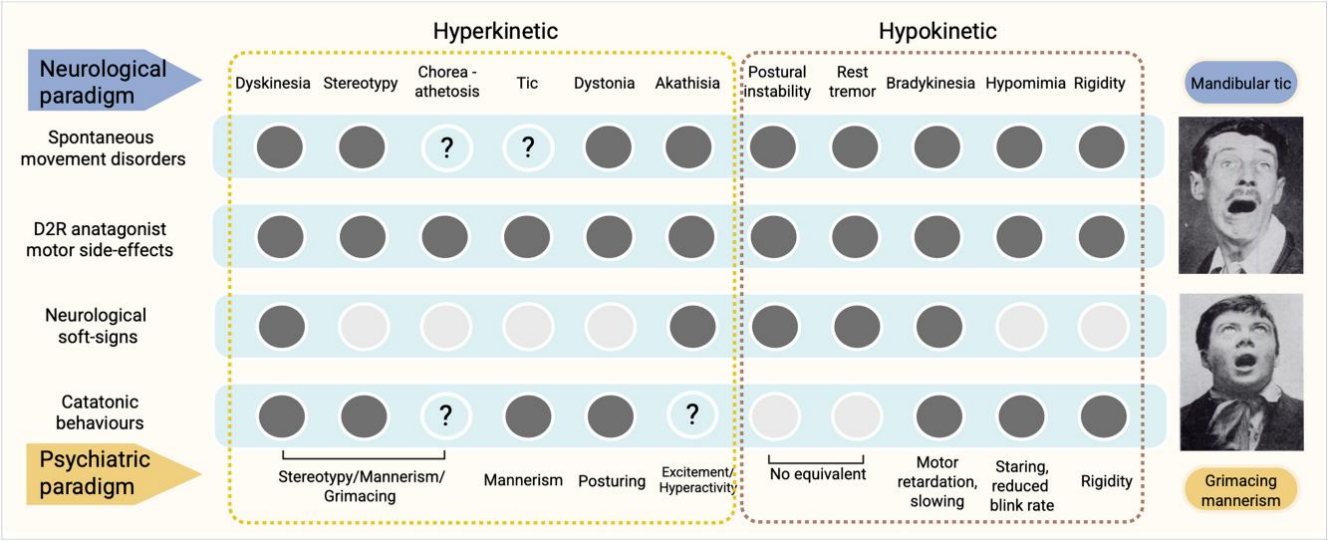
**Overlapping sensorimotor phenotypes in schizophrenia and the conflict of paradigms.** On the *top row*, sensorimotor phenotypes are given from the ‘Neurological paradigm’ and, on the *bottom row*, overlapping phenotypes from the ‘Psychiatric paradigm’.^85^ Filled circles demonstrate where an individual phenotype is described in four categories of sensorimotor disturbance seen in schizophrenia. A question mark denotes where a phenotype has not been directly assessed but where there would be reasonable grounds to suppose it may be present. Note the significant phenotypic overlap between the categories. On the *far right*, two images taken from early-mid-20th century textbooks covering topics in psychiatry (*bottom*; Norman^86^) and neurology (*top*; Wilson^87^) Similar motor phenomena are described by different vocabularies depending on whether the underlying disease is regarded as psychiatric or neurological—the ‘conflict of paradigms’.^85^ Created in BioRender. Joseph, A. (2026) https://BioRender.com/1dn8hoz.

Catatonia is a psychomotor syndrome that blurs notional boundaries separating neurological and psychiatric disease.^41^ It is distinct in the context of psychiatric conditions in that diagnosis is based predominantly upon identification of clinical signs rather than symptoms.^67^ Immobility, excessive motor activity, negativism, mutism, peculiarities of voluntary movement (posturing, stereotypies, grimacing), echopraxia (copying of actions) and echolalia (copying of speech) are amongst the signs used in current diagnostic criteria.^41,67,81^ However, at least 40 different signs related to catatonia have been reported in the literature.^67^ A particularly striking motor manifestation is catalepsy, the ability to maintain an abnormal, sometimes apparently uncomfortable, posture for extended periods of time. Waxy flexibility is a related sign where the clinician places the patient into a posture which the patient then maintains. Catatonia can be seen in a broad range of psychiatric and neurological conditions, including in the context of substance use and withdrawal, but signs of catatonia are also a diagnostic criterion for schizophrenia (alongside a range of other conditions).^37,82^ One of the challenges for classification of movement disorders in schizophrenia is that some of the motor signs associated with catatonia may be phenotypically similar to those regarded elsewhere as spontaneous movement disorders or medication side effects, even if different terms are used to describe them. For instance, the commonly used diagnostic tool, the Bush–Francis Catatonia Rating Scale,^81^ contains 23 items, three of which could be confused with signs of parkinsonism, namely rigidity, immobility and staring (with reduced blink rate), alongside stereotypies, into which category orofacial TD is included by some authorities,^36^ mannerisms, elsewhere classified as a form of tic,^83^ and posturing, which can overlap phenotypically with dystonia. Items such as ‘perseveration’ and ‘grasp reflex’^81^ can also be categorized as frontal release signs and are included in NSS assessment batteries^11^ (see later). Rogers^84^ has framed the usage of different terminology to describe often indistinguishable motor phenotypes dependent on whether the context was deemed psychiatric or neurological as a ‘conflict of paradigms’ (Fig. 1).

Complicating matters further, there is also thought to be some overlap between catatonia and neuroleptic malignant syndrome,^41^ a condition which is associated with initiation or escalation of treatment with D2R antagonists and causes rigidity, alongside dysautonomia, potentially leading to life-threatening rhabdomyolysis and malignant hyperthermia.^67^

Meta-analysis of epidemiological studies on catatonia suggests a prevalence of 9% across mental health cohorts, falling to 2.3% when only studies with greater than 1000 subjects were included.^85^ Review of UK health records estimate a prevalence of 10.1 per 100 000 patient-years.^88^ Catatonic features are also present in medication-naïve patients,^64,68,69,89^ even where diagnostic criteria for diagnosis of catatonia are not fully met.^64^ In a cohort of 200 medication-naïve participants with FEP, 31% had at least one catatonic sign, whilst only 12% fulfilled diagnostic criteria,^64^ suggesting that many patients with psychosis will exhibit catatonic features without qualifying for the full syndrome. Importantly, catatonic signs had low diagnostic specificity for schizophrenia.^64^ Further research is needed to characterize the exact relationship between catatonia and other sensorimotor features in the context of schizophrenia and other conditions with which catatonia is associated including bipolar disorder and *N*-methyl-D-aspartate receptor (NMDAR) antibody encephalitis.

### Neurological soft signs

As noted earlier, many NSSs reflect dysfunction in systems involved in sensorimotor control, coordination or planning.^2,13,15,16^ Different instruments exist to categorize and measure the severity of these signs,^12^ with the Neurological Evaluation Scale^11^ being the most frequently implemented. This scale divides NSSs into four categories: ‘sensory integration’, ‘motor coordination’, ‘sequencing of complex motor acts’, alongside ‘others’ (Table 1). These can be combined to form a composite ‘total’ score but, somewhat confusingly, some studies will also include other neurological signs (e.g. hyper-reflexia or clonus).^15,90^ Several deficits also overlap with phenotypes of dyskinesia or parkinsonism—‘fluttering’ arm movements, rest tremor, impaired rapid-alternating movements, finger tapping, postural instability—and it is worth noting that, even despite this overlap with motor disturbances typically attributed to antipsychotics, NSSs are not generally thought to be related to D2R antagonism.^15,91^ Importantly, NSS batteries may also include a smaller number of non-sensorimotor features, e.g. frontal release signs and cognitive dysfunction. Abnormalities in complex motor sequencing tasks could also indicate deficits in executive alongside motor function.

**Table 1:** Neurological soft signs: components of the neurological examination scale^11^

Sensory integration	Motor coordination	Sequencing of complex motor acts	Others
Audio–visual integration (matching tapping sounds with dots on cards) Right-left discrimination Bilateral extinction Agraphaesthesia (identify number written on tip of forefinger with eyes closed) Astereognosis (identify an object placed in hand with eyes closed)	Finger nose test (touch finger to nose with eyes closed) Rapid alternating hand movements (similar to testing for dysdiadokokinesia)^a^ Finger–thumb opposition (place digits in series on thumb) Tandem walk^a^	Fist–palm-edge test Fist-ring test (smoothly switch posture of hand between fist and a ring 15 times) Ozeretski test (place hands on table, one palm down, one in a fist, change from side to side 15 times) Rhythm tapping test (with hand reproduce a series of taps heard with eyes closed)	Frontal release signs: glabellar,^a^ palmomental, snout, suck, grasp reflexes Memory task (four-word recall task) Rest tremor^a^ Irregular ‘fluttering’ movement of extended upper limbs^a^ Romberg’s test Gaze deficits (impaired convergence, impersistence, inability to prevent head turn during eye motion testing)

^a^Signs which may also be seen in dyskinesia or parkinsonism.

Taken as a unified entity, NSSs have been demonstrated to be elevated in those with FEP,^15,89,91^ the CHR population^28,92^ and in chronic schizophrenia.^15,89,90,93^ In a meta-analysis of 204 studies quantifying associations between schizophrenia and various neurocognitive deficits, motor signs were found to have the second largest effect size of 22 domains assessed, after verbal memory impairment.^94^

Evidence is conflicting as to whether NSSs remain stable or vary over disease course in schizophrenia.^15^ In a 2014 meta-analysis, 14 of 17 studies showed improvement in NSSs, running in parallel to reduction in other symptoms, in patients treated following FEP,^95^ and this finding was corroborated by a large 1-year longitudinal study involving 349 patients.^96^ However, in a later study with 21-year follow-up, the NSS score at FEP remained highly stable in 243 patients.^89^ Elsewhere, NSS severity has been shown to increase over time in non-remitting disease courses at 2- and 5-year follow-up.^97,98^ Another large longitudinal study suggested that not all sub-domains of NSS evolve in the same way.^15^ Whilst motor coordination deficits did not vary over 10 years of follow-up,^15^ suggesting these represent a ‘trait’ marker,^15^ sensory integration signs worsened over time and in the context of clinical relapses, perhaps serving as a ‘state’ marker.^15^ This supports prior studies, reporting that sensory integration impairments fluctuate in tandem with psychotic symptoms.^95,99^ Importantly, where measured, sub-analyses suggested NSS rates are not related to D2R antagonist dosage.^15,89,95^

## Mechanisms of sensorimotor disturbance in schizophrenia

Given sensorimotor dysfunction may be an integral feature of schizophrenia, here we consider how these abnormalities, including NSSs and catatonia, arise on a mechanistic level, acknowledging that there might be multiple routes through which similar sensorimotor phenotypes may develop. Most studies reviewed in this section do not explicitly relate pathogenetic abnormalities to motor disturbances but are included to provide a broader context for current understanding of schizophrenia pathogenesis, particularly where mechanisms may be relevant to sensorimotor dysfunction. A smaller body of work has examined associations between movement disorders and brain dysfunction; however, these studies are frequently limited by small sample sizes, insufficient control for concomitant medication or illicit substance use, and reliance on clinical observation rather than objective kinematic measurement of movement abnormalities.

### Neurotransmitters: dopamine

**Figure 2:**
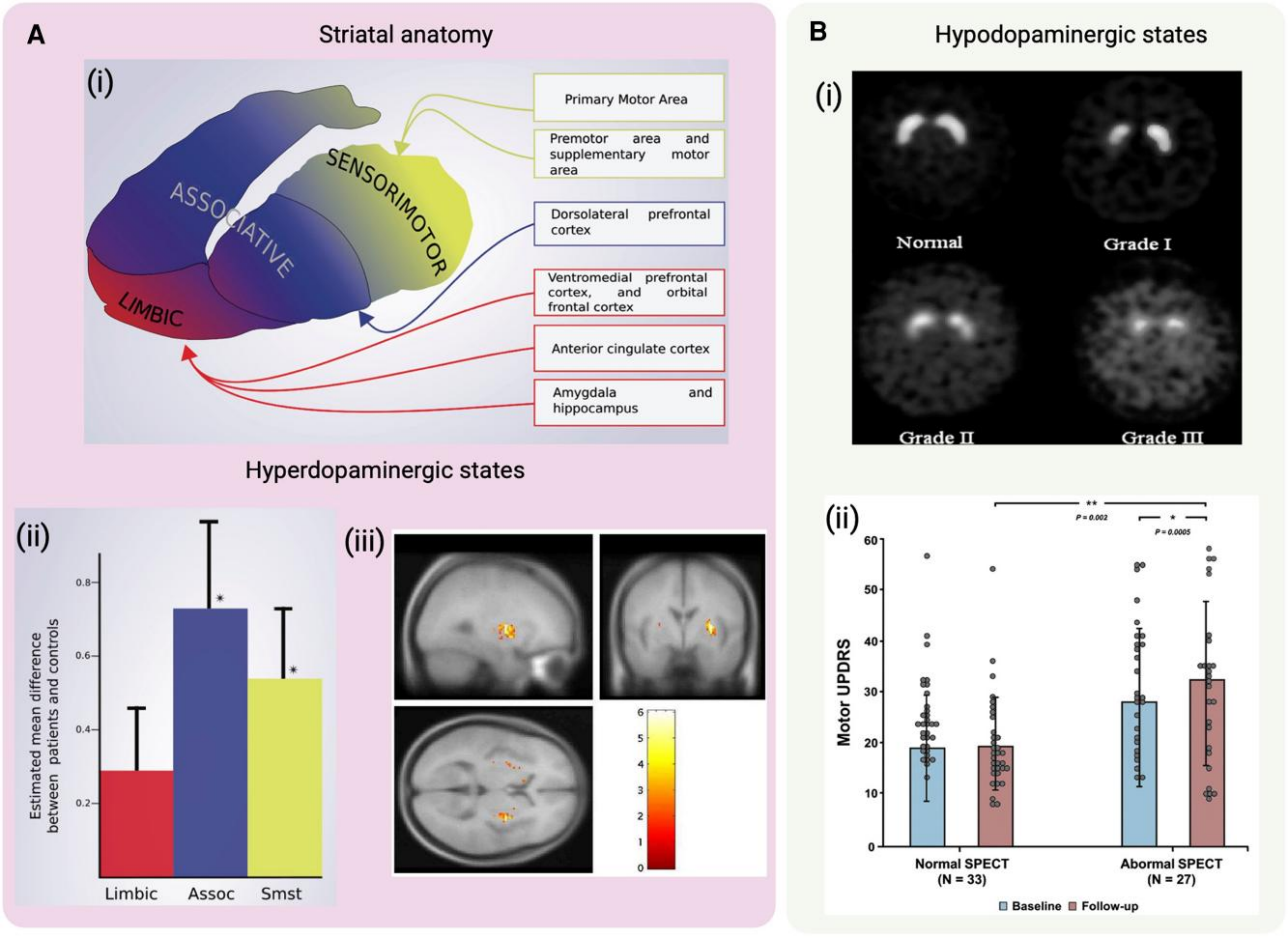
**Dopamine and the striatum in schizophrenia.** [**A**(**i**)] Functional divisions of the striatum with corresponding inputs. (**ii**) Cumulative effect sizes for elevations in dopamine synthesis or release capacity across subdivisions of the striatum, from a meta-analysis of seven PET ligand studies comparing patients with schizophrenia and controls.^106^ Panels **A**(**i** and **ii**) reproduced from McCutcheon *et al*.^106^ under the terms of Creative Commons Attribution License (CC BY 4.0). (**iii**) ^18^F-DOPA PET images demonstrate increased dopamine synthesis capacity within the sensorimotor striatum (Smst) in patients with prodromal features prior to psychosis onset compared with healthy controls.^102^ Increased dopamine synthesis in the sensorimotor striatum was most strongly correlated with development of psychosis in this cohort (reproduced with permission from Howes *et al*.^102^) [**B**(**i**)] Dopamine transporter (DaT) SPECT images from medicated patients with schizophrenia and parkinsonism and various grades of nigrostriatal denervation (Normal, Grades I–III). Reproduced from Tinazzi *et al*.^114^ with permission from Elsevier. (**ii**) Patients with schizophrenia and parkinsonism demonstrating abnormal DaT imaging exhibit progressive decline in motor symptoms of parkinsonism whilst those with normal DaT do not show progression. Reproduced from Tinazzi *et al*.^115^ with permission from Elsevier. Created in BioRender. Joseph, A. (2026) https://BioRender.com/cuogt7h. SPECT = single-photon emission computed tomography; UPDRS = Unified Parkinson's Disease Rating Scale.

**Figure 3:**
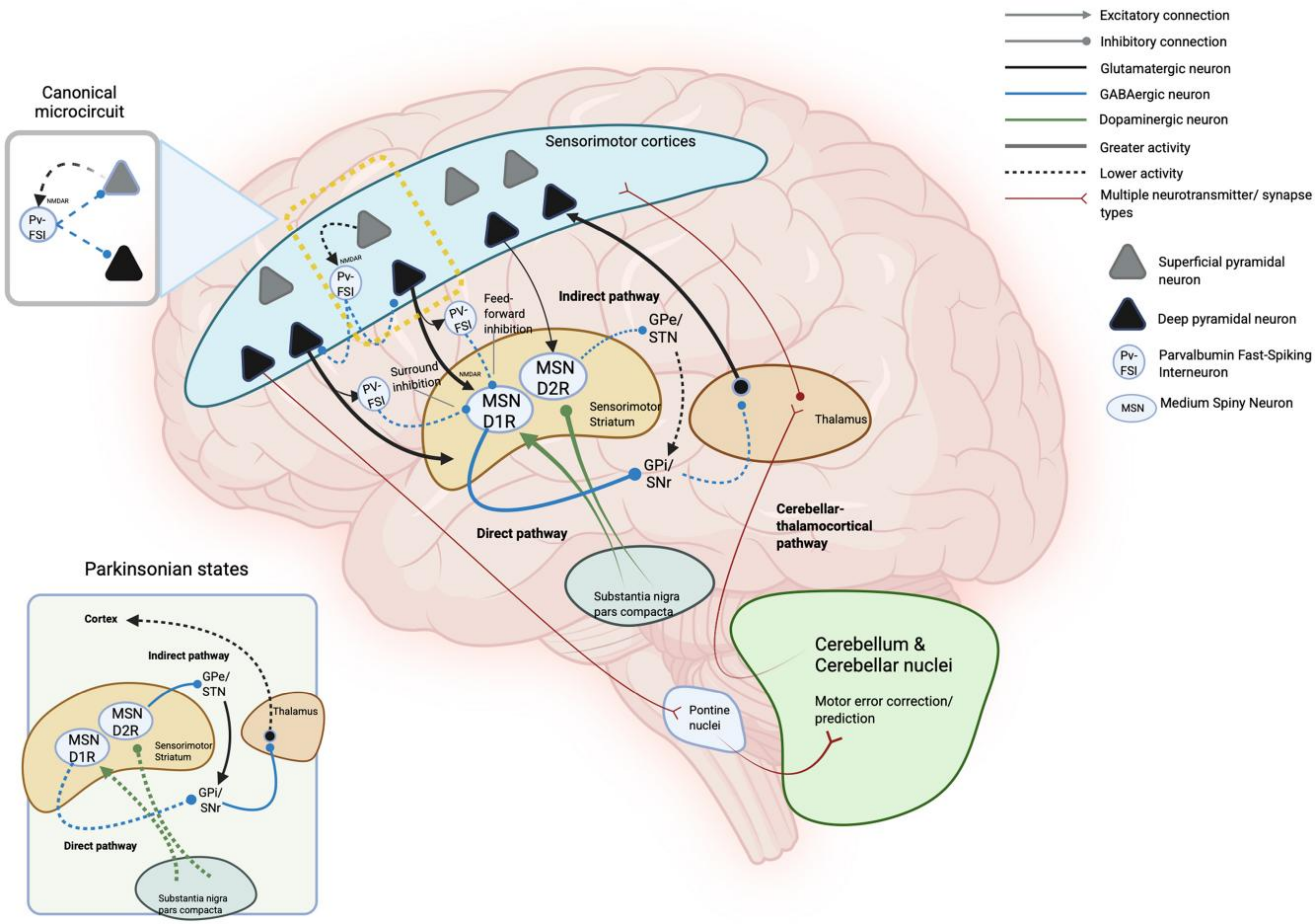
**Possible neurotransmitter and circuit disruptions leading to both hyper- and hypokinetic motor disturbance in schizophrenia.** Middle: The graphic demonstrates a variety of abnormalities which may contribute to sensorimotor disturbance in schizophrenia. Increased dopaminergic input to sensorimotor striatum from the substantia nigra pars compacta (green arrows) leads to increased direct and reduced indirect pathway activation, respectively, leading to hyperkinetic motor abnormalities. Reduced synaptic gain in superficial pyramidal cells is hypothesized to lead to motor slowing in Bayesian or predictive coding models of schizophrenia.^129^ Underactivity in GABAergic interneurons—Pv-FSIs shown in this case—is hypothesized to disinhibit glutamatergic pyramidal cells in deeper layers which project to the striatum and basal ganglia (black arrows), possibly leading to increased glutamatergic activity in these regions,^101,125^ as observed in schizophrenia^125^ and levodopa-induced dyskinesia.^130^ Glutamatergic cortico-spinal projections also synapse on Pv-FSIs which provide GABAergic inhibitory input (blue line) to principally target MSN (feedforward inhibition) and neighbouring MSN (surround inhibition). Disturbance in a cerebellar-thalamocortical pathway is implicated in motor and cognitive deficits in schizophrenia (red lines).^185^*Bottom left*: Similar model but with reduced dopaminergic input from the substantia nigra, as observed in DaT imaging of elderly patients with schizophrenia and parkinsonism.^114-116^*Top left*: Proposed primary deficit to the canonical microcircuit (vertically arranged computational units within the cortex)^127^ in schizophrenia with reduced excitability in superficial pyramidal cells leading to reduced input to inhibitory interneurons leading to corresponding pyramidal cell disinhibition.^128^ Created in BioRender. Joseph, A. (2026) https://BioRender.com/s3uxkuj. D1R/D2R = D1/D2 receptor; Dat = dopamine transporter; GPe/i = external/internal globus pallidus; MSN = medium spiny neurons; Pv-FSIs = parvalbumin-expressing fast-spiking interneurons; SNr = substantia nigra pars reticulata; STN = subthalamic nucleus.

Abnormal dopaminergic neurotransmission is a highly replicated finding in schizophrenia.^1,100,101^ Elevations in presynaptic dopamine synthesis capacity and synaptic dopamine release in the striatum are seen in FEP,^102^ schizophrenia^103^ and CHR populations,^104^ with striatal dopamine synthesis and release levels correlating with severity of positive but not negative symptoms.^101^ The striatum is segregated into ventral and dorsal components, with the dorsal being functionally subdivided into associative and sensorimotor regions [Fig. 2A(i)].^103,105^ Until recently, it was assumed that the ventral or ‘limbic’ striatum was the principal site of increased dopamine release in schizophrenia—the ‘mesolimbic hypothesis’.^103^ However, this was challenged in a meta-analysis of seven molecular neuroimaging studies comparing presynaptic dopamine synthesis and release between patients with schizophrenia and healthy controls across functional subdivisions of the striatum [Fig. 2A(ii)].^106^ Whilst increases in the ventral striatum were slight and did not reach statistical significance (*g* = 0.29, *P* = 0.09), large and significant elevations in presynaptic dopamine were seen in the dorsal striatum; in both the associative (*g* = 0.73, *P* = 0.002) and sensorimotor (*g* = 0.54, *P* = 0.005) subdivisions, with no significant differences in elevation between the two (*g* = 0.08, *P* = 0.55).^106^ Importantly, elevations in striatal dopamine release across the dorsal striatum were more pronounced in D2R antagonist-naïve than in exposed patients when compared with controls, excluding antipsychotic use as an explanation of these findings.^106^ The associative striatum comprises the caudate and pre-commissural putamen and receives projections from the dorsolateral prefrontal cortex (DLPFC),^103,105^ a structure implicated in executive control and working memory,^101,107^ but also critical for complex motor planning and action selection.^108^ The sensorimotor striatum comprises the post-commissural putamen, which receives cortical projections from the somatosensory, primary and supplementary motor cortices,^105,109,110^ and dopaminergic projections from the substantia nigra pars compacta in the ventral midbrain.^111^ Dopaminergic elevations in the sensorimotor striatum provides a mechanism through which hyperkinetic motor disturbances may arise as a primary manifestation of schizophrenia, through activation of direct and inhibition of indirect pathways, mediated through D1 and D2 receptors, respectively (Fig. 3).^110,111^ This would also explain why hyperkinetic motor disturbance seems to overlap clinically with psychotic symptoms, which also correlate with increased striatal dopaminergic activity. Notably, in unmedicated CHR youths, longitudinal follow-up shows that both apparent spontaneous dyskinesia^52^ and striatal dopamine synthesis capacity [Fig. 3A(iii)]^102,104^ rise in tandem with severity of subclinical psychotic symptoms and future risk of conversion to psychosis. Similarly, combined dopaminergic dysfunction in sensorimotor and associative striatal regions would predict that motor disturbance would overlap clinically with prefrontally mediated cognitive deficits. Indeed, both NSS and abnormal movements appear to correlate with cognitive dysfunction in CHR groups^28,53,112^ and patients with schizophrenia.^93,113^ Further work is needed to experimentally establish whether elevations in striatal dopamine release correlate with hyperkinetic motor disturbances in schizophrenia, as evidence for such an association is currently lacking.

Relatively high rates of parkinsonism recorded in unmedicated patients with schizophrenia would suggest that hypodopaminergic states also occur as a part of the disease process. Interestingly, in studies where patients (mostly elderly) with schizophrenia and parkinsonism have undergone Dopamine transporter (DaT) single-photon emission computed tomography (SPECT) imaging, evidence of nigrostriatal degeneration has been identified in a significant proportion of the 149 patients included across trials, ranging from 42% to 55% (Fig. 3D).^114-117^ Given that this imaging modality measures presynaptic dopamine transporter availability, the abnormality cannot be directly explained by postsynaptic D2R blockade. Furthermore, patients with abnormal DaT imaging were more likely to experience subsequent worsening in parkinsonian symptoms at 2-year follow-up, even after patients with changes to antipsychotic regimens were excluded from analysis,^115^ suggesting that they experience progressive loss of nigrostriatal neurons (Fig. 2B). This contrasts with data from studies in patients, not tested for parkinsonism, earlier in the disease course (including prior to D2R antagonist exposure) who do not show significant differences on DaT imaging compared with healthy controls.^118,119^ Neurotoxic effects of prolonged D2R antagonist exposure provides one explanation for observed nigrostriatal loss in patients with parkinsonism. Proposed mechanisms include oxidative stress^120^ and blockage of autophagosome production.^121^ Indeed, elderly individuals previously exposed to D2R antagonists for any reason are found to have a higher risk of developing idiopathic Parkinson’s disease than age-matched controls.^122^ However, patients with schizophrenia exhibiting parkinsonism and DaT scan abnormalities are also more likely to have asymmetric symptoms than those without,^115^ and it is not clear how medication toxicity would lead to lateralized effects. It is also possible that DaT scan abnormalities reflect a predisposition to idiopathic Parkinson’s disease, rather than a process related to schizophrenia, with clinical manifestation accelerated due to D2R antagonist exposure. Alternatively, progressive nigrostriatal degeneration may represent a primary disease process in schizophrenia, which can be exacerbated or ‘unmasked’ by D2R antagonists. This also raises the question of whether loss of nigrostriatal neurons is linked to earlier elevations in presynaptic dopaminergic activity in the sensorimotor striatum, a possibility that could be explored through longitudinal studies combing DaT and ^18^F-DOPA PET neuroimaging.

Aberrant dopamine signalling has also been used to explain how prolonged use of D2R antagonists may result in TD and other tardive phenomena.^19^ Although potential neurotoxic effects of treatment have also been proposed in this context,^3^ the dominant hypothesis posits that prolonged D2R antagonist exposure leads to compensatory postsynaptic D2R upregulation, promoting movement through an inhibitory ‘super-sensitization’ of the indirect system to dopamine.^3,110^ This mechanism could explain the transient worsening of symptoms following medication withdrawal. While rat models support this theory, showing ‘vacuous chewing’ movements associated with D2R upregulation after exposure to FGAs,^123^ human studies provide more limited evidence. Meta-analyses of ligand neuroimaging^118,124^ and post-mortem studies^124^ demonstrate significant yet only small and inconsistent elevations in D2/3R expression in patients with schizophrenia, including those exposed to D2R antagonists. Furthermore, genome-wide association studies reveal that only a subset of TD risk-associated loci relate to dopaminergic function or drug metabolism,^3,101,110^ indicating that the super-sensitization hypothesis may be oversimplistic.

### Neurotransmitters: glutamate and GABA

Data from genetic, post-mortem and *in vivo* neuroimaging studies now support the notion that abnormalities in glutamatergic and GABAergic neurotransmission are central to schizophrenia pathogenesis.^101,125,126^ These may also contribute to associated sensorimotor disturbances through several possible mechanisms.

Glutamatergic pyramidal cells are the primary excitatory neurons in the cortex, comprising approximately 80% of cortical cells.^127^ Recent neural network modelling of electroencephalographic changes observed across different paradigms indicate that reduced synaptic gain (excitability) in superficial pyramidal cells may represent a fundamental deficit in schizophrenia (Fig. 3),^128^ proposed as a substrate for motor slowing within predictive coding accounts of psychosis (see later).^129^ Interestingly, neuroimaging studies appear to corroborate computational modelling findings, showing reduced glutamate and related metabolite levels in the frontal cortex of patients with schizophrenia compared with controls, alongside elevations within the basal ganglia that correlate with psychotic symptom severity.^125^ Further work in this area is warranted, given the difficulties of relating regional neurotransmitter differences in ligand-based studies to underlying neurocircuitry, as well as the heterogeneity of both findings and study populations across relevant trials. Nevertheless, it is worth noting that, in idiopathic Parkinson’s disease, levodopa-induced dyskinesia has been linked to increased glutamatergic NMDA receptor activation within the striatum,^130^ a finding supported by animal models,^131^ suggesting that investigation of the interplay between glutamatergic activity and sensorimotor disturbance in schizophrenia may represent a fruitful direction for future research.

McCutcheon *et al*.^1^ have also hypothesized that reduced excitability in cortical superficial pyramidal cells and increased striatal glutamatergic and dopaminergic activity may be interconnected phenomena in schizophrenia, proposing that reduced activation of inhibitory interneurons by superficial pyramidal cells, leads to disinhibition of counterparts within deeper cortical layers which extend glutamatergic projections to the striatum, facilitating increased dopamine release from nigrostriatal inputs.^1^ Whilst direct evidence for this is lacking, supporting data include negative correlations between markers of prefrontal glutamatergic activity and both striatal dopaminergic activity^132^ and severity of psychotic symptoms,^125^ as well as studies in rats showing that optogenetic stimulation of glutamatergic infralimbic cortico-striatal projections leads to increased dopamine release in the nucleus accumbens.^133^ This model is particularly relevant to sensorimotor dysfunction since it could provide a mechanism through which a circuit level deficit—reduced excitability in glutamatergic neurons within the superficial cortex—could lead to both hypokinetic (motor slowing) and hyperkinetic motor disturbances via modulation of direct and indirect basal ganglia pathways. Supporting this possibility, ketamine, an NMDAR antagonist believed to reduce cortical glutamatergic neurotransmission,^101^ mimics negative psychotic symptoms^134^ whilst also driving increases in striatal dopamine synthesis^135^ and inducing a range of motor disorders observed in schizophrenia, including slowed ocular smooth pursuit,^136^ catalepsy,^137^ abnormal repetitive movements^138^ and excessive tongue movements in rats, which serve as an animal model of TD.^139^

Recent data from post-mortem studies^140^ and computational modelling of electroencephalographic data^128^ also indicates underactivity of inhibitory GABAergic interneurons in schizophrenia, due to reduced NMDAR-mediated glutamatergic inputs from pyramidal cells. These include parvalbumin-expressing fast-spiking interneurons (PV-FSIs), which play an important role in motor control,^110^ providing feedforward inhibition to striatal medium spiny neurons after receiving excitatory input from cortico-striatal projections^141^ and providing surround inhibition necessary for motor choice execution (Fig. 3).^142^ Relevantly, selective lesioning of these neurons leads to dyskinesias, though only in rodent models.^143^ Pv-FSIs also coordinate rapid firing patterns of pyramidal cells,^140^ generating electrographic high-frequency gamma-band activity^144^ which is disrupted in schizophrenia^145^ and shown to lead to deficits in working memory.^145,146^ Gamma-band disruption has been recorded in both motor and cerebellar cortices during motor tasks, in a small sample of 12 adolescent patients with a diagnosis of early-onset psychosis rather than schizophrenia^144^ and further neurophysiological work is required to detect whether this abnormality plays a causal role in sensorimotor deficits. Interestingly, in a study using paired-pulse transcranial magnetic stimulation (TMS) to compare patients with schizophrenia with (*n* = 60) or without (*n* = 23) psychomotor slowing and healthy controls (*n* = 40), markers of reduced cortical inhibition, and by extension GABA-A dysfunction, were shown to be associated with impaired motor coordination and aberrant functional connectivity between motor regions during functional MRI (fMRI),^147^ alongside psychomotor slowing in patients with higher scores on a catatonia rating scale.^147^ Importantly, whilst significant associations remained when medication treatment was included as a covariate, it is possible that treatments could have affected neurophysiological results. Furthermore, markers of lowered cortical inhibition used in the study such as reduced short interval intracortical inhibition (SICI) to motor-evoked potential ratio are also seen in hyperkinetic movement disorders,^148^ which is difficult to reconcile with the apparent association with psychomotor slowing and limits the presumed specificity of the finding. The authors suggest that it may relate to a state in which there is in effect too much ‘noise’ in the sensorimotor system,^147^ citing alternate studies in which reduced SICI was observed during motor tasks in patients with schizophrenia^149,150^; in one case correlating with increased force and electromyographic variability in a grip task,^149^ however, importantly in another showing no association with impaired performance in a stop-start signal protocol.^150^ This possibility is of interest given computational accounts of schizophrenia in which psychomotor slowing arises due to impaired integration of sensory data into predictive models required for motor action (see later). However, it is also worth noting that in some cases, reduced SICI has been observed in another hypokinetic motor disorder, Parkinson’s disease,^148,151^ (including in the absence of dyskinesia),^151^ highlighting the importance of further work determining the specificity of neurophysiological findings to the spectrum of motor disturbances observed in schizophrenia.

Abnormal synaptic neuroplasticity between Pv-FSIs and medium spiny neurons has also been hypothesized to contribute to tardive and spontaneous dyskinesias,^110^ given abnormal synaptic plasticity is observed in healthy individuals exposed to D2R antagonists^152^ and a variety of movement disorders including Huntington’s disease^153^ and levodopa-induced dyskinesia.^154^ However, the ubiquity of abnormal synaptic plasticity across neuropsychiatric conditions may again also limit the presumed specificity of this process to sensorimotor disturbance in schizophrenia.

Future research could examine how alterations in neurotransmitter release drive sensorimotor disturbance and correlated electrophysiological abnormalities across relevant microcircuits and wider brain networks, including neurotransmitters implicated in schizophrenia and sensorimotor dysfunction but not described here, such as serotonin and acetylcholine.

## Computational models of sensorimotor disturbance in schizophrenia

Sensorimotor signs have already been incorporated into computational models of schizophrenia, particularly in the domain of ‘predictive coding’.^129,155^ Briefly, ‘predictive coding’, a Bayesian account of neural computation, assumes a nervous system which generates and continually updates an internal predictive model of the world.^129,156^ Sensory information is constantly compared against prior beliefs (or predictions) and, where mismatch occurs, creates prediction errors, which in turn may cause updating of predictions. Underpinning this is the idea that the nervous system is made up of multiple intercommunicating levels both in respect to its structure and information processing architecture, arranged in a vertical ‘hierarchy’. This means that higher order centres (e.g. cortical structures) send ‘top-down’ prior beliefs which are contrasted against ‘bottom-up’ information, which originate from lower layers of the hierarchy (e.g. afferent sensory neurons) to create prediction errors.^156^ Broadly, the aim is to minimize prediction error.^156^ One way of doing this is by refining the agent’s model of the environment (perception), but we can also interact with the world (action) in ways that fulfil our expectations (known as ‘active inference’).^157^

**Figure 4:**
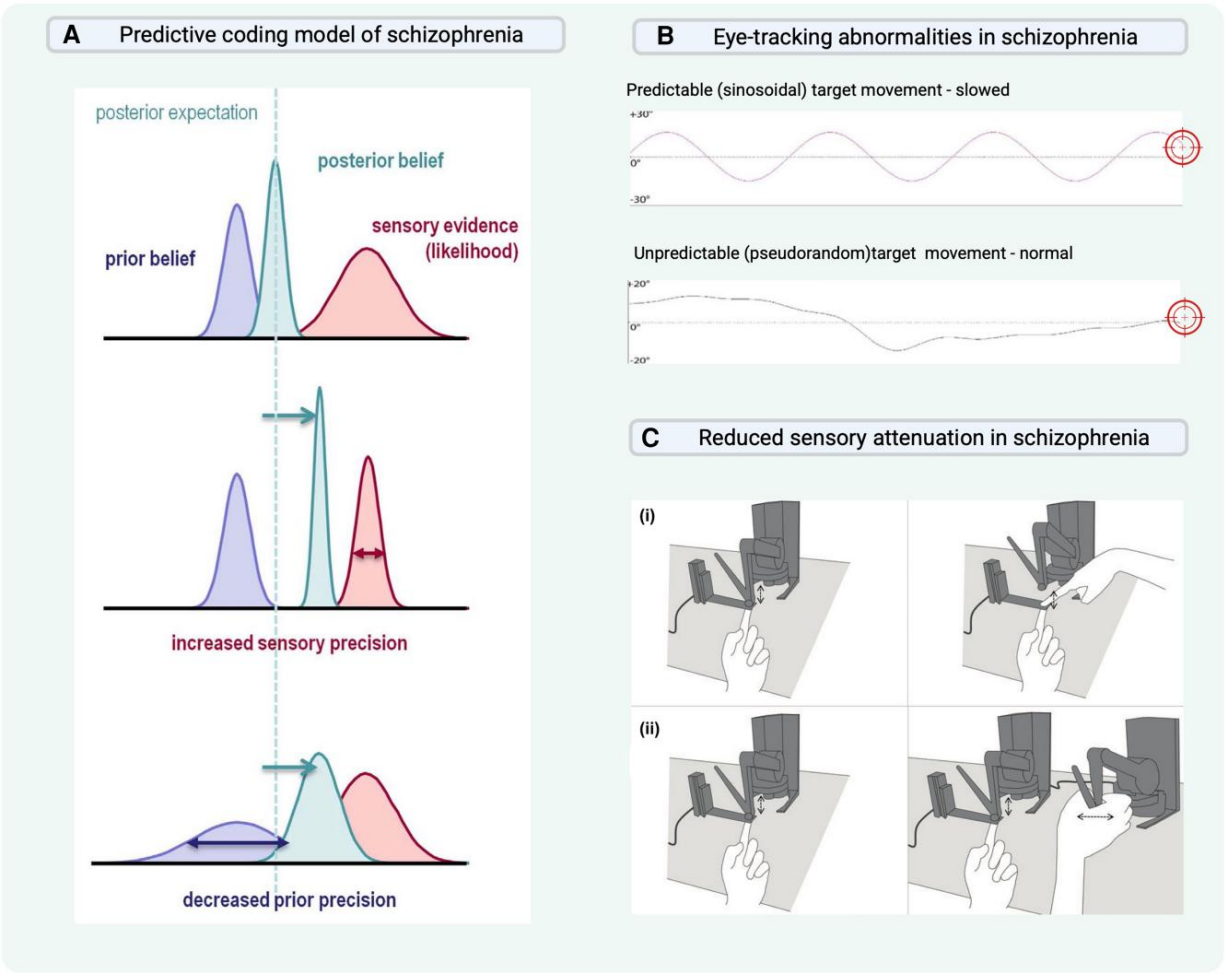
**Bayesian model of schizophrenia and two associated sensorimotor abnormalities.** (**A**) Proposed Bayesian model of schizophrenia. Graphs depict Gaussian probability distributions representing prior beliefs, posterior beliefs and the likelihood of sensory evidence related to some variable. The dashed line indicates the posterior expectation, while the width of the distributions reflects their variance. Precision, which is the inverse of variance, significantly impacts posterior beliefs. Essentially, the posterior belief is influenced by prior and sensory evidence based on their relative precision (*top*). This means that increasing sensory precision (*middle*) or decreasing prior precision (*bottom*) can bias the posterior expectation towards sensory data. This is thought to explain sensorimotor abnormalities in schizophrenia shown on the *right*. Reproduced from Adams *et al*.^129^ under the terms of the Creative Commons Attribution License (CC-BY). (**B**) Eye-tracking experiments in patients with schizophrenia demonstrate slowed following of targets which follow a predictable pattern compared with controls but normal tracking of a target moving in a pseudorandom fashion, i.e. impaired utilization of prior belief (or predictions) but retained ability to respond to sensory evidence. Reproduced from Nkam *et al*.^161^ with permission from Elsevier. (**C**) Sensory attenuation can be measured using the force-matching task, where participants match a reference force either by pressing directly on their finger (**i**) or using a robotic lever (**ii**). Healthy individuals typically exert more pressure when pressing on themselves compared with using the robot, indicating sensory attenuation. This phenomenon may occur because sensory attenuation reduces the intensity of sensory evidence from self-generated actions. In contrast, patients with schizophrenia show less pronounced differences in force-matching, as their sensory attenuation is reduced, leading to a more accurate estimation of self-generated forces.^156,165^ Reproduced with permission from Pareés *et al*.^264^ Created in BioRender. Joseph, A. (2026) https://BioRender.com/ta16q3i.

Whilst a complete account is beyond the scope of this review, the hypothesized disturbance in schizophrenia from a predictive processing perspective relates to the concept of ‘precision’: the degree of certainty (inverse variance) attributed to the probabilistic encoding of information.^129^ Specifically, it is thought that in schizophrenia, greater precision or confidence is allocated to ‘bottom-up’ sensory information, and less to ‘priors’ or ‘top-down’ predictions (Fig. 4A).^158^ Adams *et al*.^129^ have linked this to some of the neurophysiological disturbances described earlier, particularly hypofunction of pyramidal cells, NMDARs and inhibitory interneurons. A helpful example of this is in a specific case of motor slowing, namely in smooth pursuit eye movements, which show reduced ‘gain’ (transformation of the retinal error signal into oculomotor drive) both in patients with schizophrenia and non-affected relatives.^159,160^ Importantly however, tracking speed reverts to normal if a target object moves in a pseudorandom fashion, only slowing if movement becomes predictable (for instance a sinusoidal wave),^161^ suggesting that the motor deficit is in the predictive power of ‘prior beliefs’ about target motion (Fig. 4B),^162^ although evidence from target occlusion experiments is more controversial.^163^

Another interesting sensorimotor abnormality, which is thought to contribute to motor disturbance in schizophrenia,^129^ is reduction of sensory attenuation—the lower intensity that sensory consequences of our own movements typically have^156^ (illustrated by the fact that self-induced sensations are not typically perceived as ticklish).^164^ Brown *et al*.^156^ provided a computational account of this, where the increased precision attributed to sensory information in schizophrenia makes it more difficult to distinguish self-generated versus externally generated sensations. Sensory attenuation can be experimentally quantified using the ‘force matching’ task (Fig. 4C), in which subjects are asked to mimic a reference force, either by pressing on themselves directly, or by using a robotic lever to match the perceived pressure.^165^ While healthy people generate excessive pressure when pressing on themselves as opposed to more accurate pressure when using the robot, this difference is less pronounced in patients with schizophrenia.^156,165^ This is because in healthy controls, sensory attenuation of self-produced pressure sensations makes one push harder to match a reference force, but when sensory attenuation is reduced (in schizophrenia), the self-produced force is more accurate.

Active inference modelling implies that sensory attenuation is not just an attentional phenomenon but may facilitate movement.^157^ Assuming the brain is a predictive coding hierarchy, when it wants to move, predictions about the current position of the body will clash with predictions about its future position. The latter can be fulfilled by reducing the relative precision of the former, i.e. attenuating current sensory input.^156^ For example, if you are pushed forward, ‘bottom-up’ proprioceptive signals will generate prediction errors against the ‘prior belief’ that you are standing still, which will (hopefully) lead to the activation of a postural reflex arc to keep you upright. When you want to move forward, however, you must somehow suppress (or attenuate) this ‘bottom-up’ sensory data to reduce prediction error and thereby inhibit activation of compensatory reflexes. Loss of sensory attenuation could therefore impair movement, as in catatonia, and explain passivity or perceived loss of agency, assuming that inferences about agency for movements are based on the accurate prediction and attenuation of their sensory consequences.^129^ Supporting this possibility, when humanoid robots are programmed so that the ‘precision’ of top-down signals is decreased, they also exhibit catatonia-like behaviours including motor disorganization and posturing.^166^ Interestingly, resting state fMRI research also shows a positive correlation of connectivity between the thalamus and sensory cortex and severity of dyskinesia and catatonic behaviours in schizophrenia.^93^ Importantly, the thalamus is the main structure responsible for feedback relay to the cortex; hence, it is likely involved in propagating proprioceptive prediction errors towards hierarchically higher structures, a process that is modulated by suppression according to the predictive coding model of sensory attenuation.^129^ Furthermore, in idiopathic Parkinson’s disease, degree of impairment in sensory attenuation correlates positively with severity of motor symptoms and negatively with levodopa dose, suggesting that in schizophrenia, altered sensory attenuation may also be related to both striatal dopaminergic dysfunction and parkinsonian symptoms.^167^ Nevertheless, the presence of abnormal sensory attenuation in idiopathic Parkinson’s disease and other neuropsychiatric conditions, including functional neurological disorder, also further emphasizes the need to critically evaluate the specificity of sensorimotor abnormalities to schizophrenia.

The development of computationally informed testable hypotheses for how other sensorimotor disturbances develop in schizophrenia is an important area for further research, especially given that predictive coding techniques show promise in modelling various neurological motor signs such as hyper-reflexia, ataxia and intention tremor.^157^

### The neurodevelopmental hypothesis and sensorimotor disturbance

**Figure 5:**
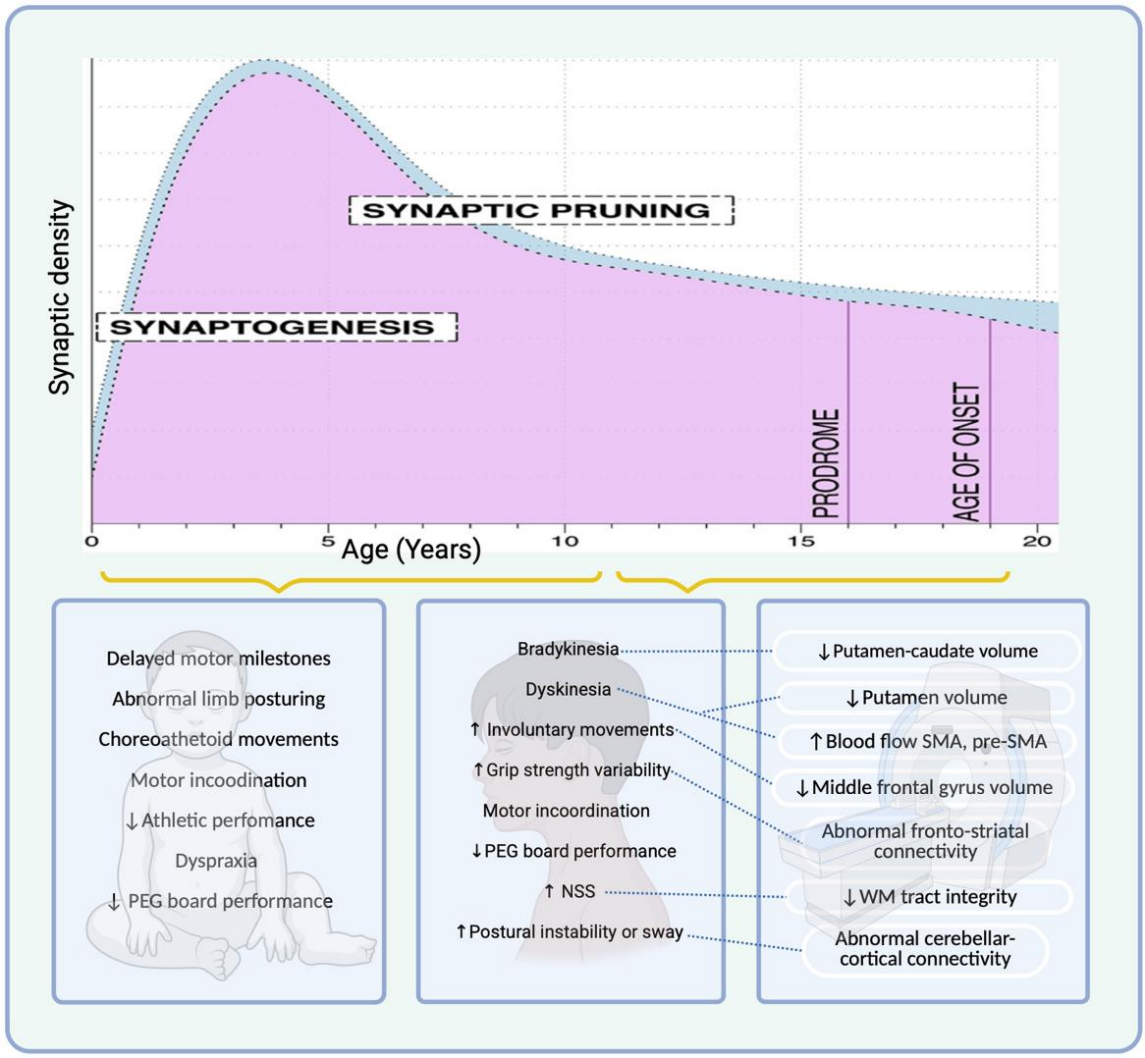
**Possible neurodevelopmental differences in children and adolescents at risk of developing schizophrenia.**  *Top*: Theoretical difference in synaptic density across periods of synaptogenesis and pruning in children and adolescents who go on to develop schizophrenia (dark pink) versus those not at risk (light blue). Reproduced from Howes and Shatalina,^169^ under the terms of the Creative Commons Attribution License (CC BY 4.0). *Bottom left*: Motor abnormalities in children who develop schizophrenia in later life. *Bottom middle*: Motor abnormalities in clinically high risk (CHR) adolescents. *Bottom right*: Structural and functional MRI changes associated with motor abnormalities in CHR adolescents. Created in BioRender. Joseph, A. (2026) https://BioRender.com/sq43iff. NSS = neurological soft signs; SMA = supplementary motor area; WM = white matter.

Abnormalities in neurodevelopment are central to many current theories of schizophrenia pathogenesis.^168,169^ Neuroimaging studies demonstrate that brain structural differences relative to healthy controls are already present both in FEP and in adolescents with prodromal symptoms.^168,169^ Literature on sensorimotor disturbance in children and adolescents who later develop schizophrenia could also be seen to lend credence to the ‘neurodevelopmental hypothesis’, suggesting that subtle neurological abnormalities are present long before psychotic symptoms develop (Fig. 5). On retrospective review of childhood video footage, adults with schizophrenia demonstrated elevated rates of choreoathetoid movements and abnormal limb posturing before the age of 2 years, when compared with age-matched controls or unaffected siblings.^170^ Children who exhibited these motor disturbances were also more likely to exhibit ventriculomegaly in adulthood,^171^ an imaging finding associated with schizophrenia.^103^ In a large prospective birth cohort study, increased rates of motor incoordination and abnormal involuntary movements were observed in children who later developed schizophrenia, compared with their unaffected siblings.^172^ Incremental delays in motor milestones^173,174^ and poor athletic performance at school^175^ have both also been reported to correlate positively with the risk of psychosis in later life. In adolescents, elevated rates of both NSSs^92^ and spontaneous movement disorders^29,53,55^ are described during the prodromal CHR period (Fig. 5). Importantly however, establishing a causal relationship between neuromotor developmental abnormalities and the later emergence of schizophrenia remains challenging. Such abnormalities may instead reflect alternative risk factors, including socioeconomic deprivation, adverse life events or co-occurring developmental conditions that independently increase vulnerability to schizophrenia (see later). Moreover, most children with neuromotor abnormalities do not develop schizophrenia, and not all adults with schizophrenia show evidence of early motor dysfunction. This asymmetry raises questions about the specificity of the neurodevelopmental hypothesis and highlights sensorimotor dysfunction as a potentially useful framework for further investigation. It also remains unclear whether motor abnormalities are uniquely associated with the development of psychosis. For example, in the Adolescent Brain Cognitive Development (ABCD) study involving 11 878 children aged 9–11 years, motor developmental delays, incoordination and psychomotor agitation were each associated with psychotic-like experiences, but these features also correlated significantly with measures of depression.^176^

Childhood and adolescence are stages of development in which the brain undergoes dramatic changes in the balance between synaptic proliferation and loss.^169^ Synaptogenesis and synaptic density peak at around age 5 years, followed by an extended period of synaptic pruning which continues into late adolescence and early adulthood (Fig. 5).^169,177,178^ Imbalance between synaptic generation and pruning in schizophrenia with subsequent reduction of synaptic density^169^ is supported by post-mortem investigations^179^ and evidence of altered gyrification and decreased cortical grey matter volume from neuroimaging studies.^169^ Such reductions in synaptic density appear to progressively evolve during adolescence,^169^ and may explain motoric abnormalities seen in CHR stages. Structural neuroimaging findings show that apparent bradykinesia in unmedicated CHR adolescents is correlated with reduced ipsilateral putamen and bilateral caudate volumes,^29^ and abnormal involuntary movements with volume reduction of the middle frontal gyrus^180^ and striatum.^53^ Interestingly, the temporal course of cortical development follows a caudo-rostral gradient (earlier maturation of visual, sensory and motor regions followed by later maturation of more frontal regions, including the DLPFC),^169,181^ which may explain why sensorimotor abnormalities can occur prior to psychotic symptom onset. In CHR adolescents, increases in overall burden of NSSs,^92^ motor incoordination,^112^ motor sequencing abnormalities,^112^ variability in grip strength^182^ and rates of dyskinesia^52^ have all been shown to correlate with an elevated risk of future conversion to psychosis. The predominant occurrence of dyskinesia in the lower face, followed by hands and fingers in some studies, could also be related to synaptic density alterations, given that these areas receive the most extensive somatotopic representation in the motor cortex^183^ and are therefore perhaps more vulnerable to stochastic failures of synaptic development. Notably, another neurodevelopmental disorder, Tourette syndrome, is also primarily associated with motor symptoms involving the face and neck.^184^

Alongside structural abnormalities, alterations in functional connectivity also relate to sensorimotor disturbance in the developing brain of people at risk of schizophrenia (Fig. 5). In unmedicated CHR adolescents, dyskinetic movements correlate with fMRI markers of increased blood flow to premotor and supplementary motor areas,^180^ and grip force variability is found to be related to impaired connectivity between the DLPFC and the striatum.^182^ Furthermore, postural instability, as measured by degree of sway, is also elevated in CHR adolescents and correlates with resting-state dysfunctional connectivity between cerebellar and frontal cortices.^185^ Similarly, in the ABCD trial, whilst aberrant cortico-striatal connectivity was observed in adolescents with both depression and psychotic-like experiences, dysfunctional cortico-cerebellar connectivity was only associated with the latter.^176^ Dean *et al*.^112^ also observed greater functional connectivity between the thalamus and cortical sensorimotor areas in CHR participants with higher rates of motor impairment.

Importantly, delayed motor milestones and sensorimotor dysfunction in youth are also seen in other neurodevelopmental conditions suggesting that neuromotor developmental abnormalities may not be specific to schizophrenia and psychosis. However, risk of schizophrenia or psychosis is also elevated in some of these conditions. For instance, patients with autism spectrum disorder (ASD) exhibit elevated rates of motor deficits,^186^ impaired praxis^187^ and NSSs^188^ compared with neurotypical children and ASD is associated with a 3–4-fold increased risk of psychosis,^189^ elevated psychotic-like experiences in youth^189^ and shares overlapping genetic risk with schizophrenia.^190^ Childhood dyspraxia, another common neurodevelopmental condition involving motor disturbance,^191^ has also been shown to be associated with elevated psychosis risk in later life in a prospective study of 244 10–13 year olds (though importantly in a population made up principally of first-degree relatives of individuals with schizophrenia or other psychiatric diagnoses),^191^ and signs of dyspraxia have been shown to occur in ∼25% of adults with schizophrenia.^192^ Unfortunately, as studies evaluating correlations between sensorimotor dysfunction in youth and subsequent development of psychosis tend not to report on other co-existent neurodevelopmental conditions, overlap between schizophrenia risk and conditions such as dyspraxia and ASD cannot be fully ascertained. This is a critical area for further investigation.

### Structural and functional correlates of sensorimotor dysfunction in adulthood

As for psychiatric symptoms, abnormalities in brain morphology and functional connectivity also appear to be associated with sensorimotor abnormalities observed in adults with schizophrenia. In a meta-analysis implementing activation likelihood to link data from 21 neuroimaging studies with results of NSS test batteries, NSS scores correlated with reductions in grey matter volume in the precentral gyrus, inferior frontal gyrus, thalamus and cerebellum.^13^ Their findings support previous observations that people with schizophrenia have a smaller cerebellum compared with controls, and that cerebellar atrophy is associated both with burden of NSSs and motor incoordination.^193,194^ Altered morphology in the putamen, globus pallidus and caudate nucleus have also been found to correlate with NSS in separate studies,^195,196^ as have alterations in white matter tract integrity across multiple regions.^196,197^ Abnormal connectivity in cerebellar-cortical networks on resting state fMRI of adult patients with schizophrenia also appear to correlate with overall burden of NSSs,^198^ impairment in finger-tapping tasks^199,200^ and increased postural sway.^199^

Interestingly, observed associations between cerebellar dysfunction and sensorimotor deficits lend some support for the ‘cognitive dysmetria’ hypothesis,^155,201^ which proposes that symptoms in schizophrenia emerge through a failure to create accurate internal predictions about the world. As for motor dysmetria, a neurological sign in which a failure to predict distance leads to over- or undershooting a given target, ‘cognitive dysmetria’ is thought to be driven in part by dysfunction in the cerebellum and its wider connections to the frontal lobes and basal ganglia.^201^ That cerebellar dysfunction is also implicated in sensorimotor disturbance in schizophrenia, suggests that the ‘dysmetria hypothesis’ has relevance beyond cognitive and perceptual domains, and may provide a unifying account for how both motor and non-motor symptoms arise. This highlights the importance of further research into cerebellar dysfunction in schizophrenia and its motor correlates.

Studies examining structural correlates of abnormal involuntary movements in unmedicated patients with first FEP or schizophrenia are unfortunately limited. In a study of unmedicated Indian patients with chronic schizophrenia, those with dyskinesia exhibited enlarged left lentiform nuclei, while non-dyskinetic patients had higher lateral ventricle-hemisphere ratios.^202^ However, the limited sample size affects the reliability of these comparisons. In medicated patients, studies on the structural correlates of TD have yielded mixed results, including volume reductions in the caudate nucleus,^203,204^ and increases in both the globus pallidus and putamen.^196,205^ TD has also been shown to be associated with cortical atrophy, ventricular enlargement^196,206^ and reduced white matter tract integrity across the DLPFC, mesial frontal, somatosensory and temporal cortices.^207^ In addition, elevated NSSs, abnormal involuntary movements and dyskinesia appear to be correlated with increased resting state functional connectivity between selected areas within the sensorimotor cortex, thalamus, subthalamic nucleus and cerebellum.^93^

Catatonia is also associated with structural changes on brain imaging in patients with schizophrenia.^208^ These include alterations in cortical thickness and local gyrification in several brain areas including within the premotor, motor and parietal cortices,^209,210^ alongside white matter abnormalities in motor tracts.^211,212^ FMRI studies in patients with schizophrenia also suggest a relationship between catatonia and elevated blood perfusion in bilateral supplementary motor areas,^213^ aligning with case studies following lobotomy and other frontal lesions, alongside non-human primate experiments, which implicate supplementary motor area dysfunction in the development of waxy flexibility and negativism.^208,214^ Hyperconnectivity between the thalamus and cortical motor areas has also been associated with catatonia alongside dyskinesia.^93^

## Sensorimotor system in schizophrenia: opportunities for clinical practice and research

In this section, we consider opportunities that might arise from better characterization and understanding of sensorimotor signs and symptoms in schizophrenia. Sensorimotor features are especially amenable to instrumental assessment. Whilst detection, rating and monitoring of positive and negative symptoms often demands protracted medical interviews or self-completed scales, mechanical or computational sensorimotor assessments could serve as cheaper, more reliable alternatives, designed with improved patient tolerability in mind.^16^ Advances in wearable devices, smartphone-enabled motor sensors and automated video analysis are already being implemented to improve early detection and symptom tracking in neurological practice, not only in common movement disorders like Parkinson’s disease,^215,216^ but also in conditions traditionally regarded as pure cognitive disorders, for example Alzheimer’s disease.^217^ These tools could have a range of benefits, explored later in the domains of research and clinical practice (Table 2).

**Table 2:** Instrumental assessment of sensorimotor disturbances in schizophrenia

Sensorimotor sign	Instrumental measure	Methodology
Dyskinesia	Increased force variability (arm, tongue, grip)	Measures variability in applied force whilst subject applies constant pressure to strain gauge with hand or finger,^56,219,237^ tongue^221^ or grip.^182^ Tremor excluded by filtering techniques.
Tremor	Tremor	Similar to the above, filtered tremor waveform undergoes Fourier transform to identify frequency range of interest (e.g. 4–6 Hz parkinsonian tremor).^56^
Bradykinesia	Decreased velocity scaling	Measures movement speed between fixed points. Bradykinetic patients show difficulty increasing velocity when target distance expands, revealing impaired motor scaling ability.^56,219^
Rigidity	Decreased passive displacement	Measures force required to passively displace test-limb using a motor driven beam or platform. Effects of synkinetic movements of contralateral limb distinguish rigidity from peripheral muscle stiffness.^265^
Postural instability	Increased postural sway	Motion capture systems tracks movement of the body’s centre of pressure during quiet standing on a force platform.^185,266,267^
Multiple motor signs	Locomotor activity	Ambulatory locomotor activity measured using wrist-worn piezoelectric accelerometer (actigraph) can identify patients with drug-induced akathisia and pseudoakathisia.^246^ Reduced activity is associated with dystonia, parkinsonism and catatonia.^268,269^
	Altered handwriting kinematics	Digital handwriting analysis software measures kinematic features like reduced size (micrographia) or velocity (suggesting parkinsonism) and dysfluency (dyskinesia) from tablet-based tasks.^29,182,243,244^
	Motion capture technology	Cameras record subject performing an upper limb or facial motor task which is then analysed using motion capture technology to quantify variables relating to dyskinesia or bradykinesia.^222^
Gait disturbance	Motion capture technology	Gait recorded on camera, motion capture technology quantifies parameters such as stride length, velocity, gait variability, posture and coordination of right/left sided movement. Reduced stride length, cadence and velocity can be used as a surrogate for psychomotor slowing.^234^ Gait variability and ‘hanging’ head posture particularly associated with schizophrenia and NSSs.^234,270^
	Footswitch system	Measures various gait parameters through digitally enabled sensors implanted in insoles.^233^

### Diagnostics

It has been suggested elsewhere that instrumentally detected sensorimotor abnormalities could be used to differentiate schizophrenia from other psychiatric conditions.^218^ Instrumental assessment has been shown to be more able to detect both spontaneous dyskinesia and parkinsonism than clinical rating scales in unmedicated patients with FEP,^219^ giving estimated prevalences of 13%–20% and 18%–28%, respectively.^219^ These prevalence rates approach requirements for a DSM ‘Group A’ criterion (30%–40%), needed to make a formal diagnosis of schizophrenia,^218,219^ and are roughly comparable to the prevalence of thought disorder or catatonic behaviours.^218^ Further research is required to compare the diagnostic value of motor features against current criteria. Of note, dyskinesia in these studies was measured through assessment of force-variability in the upper limb.^219,220^ Given that in schizophrenia, dyskinesia is predominantly present in the lower face and jaw,^3,7^ available devices designed to quantify lingual force-variability,^221^ or automated video analysis of facial movements^222^ or speech might be expected to exhibit even higher levels of diagnostic sensitivity.

Utility of NSS in the early diagnosis of schizophrenia might be limited given they are also present in psychiatric conditions with overlapping symptomatology such as bipolar disorder^70^ and autism spectrum disorder.^223^ Zhao *et al*.^188^ were able to demonstrate that NSS scores effectively differentiated schizophrenia from major depressive disorder but not bipolar disorders. Peralta *et al*.^89^ found that although in FEP, higher neuromotor dysfunction scores combining NSS, spontaneous movement disorder and catatonic features were associated with greater likelihood of receiving an eventual diagnosis of schizophrenia, motor dysfunction had been evident in ∼15% of patients who developed bipolar disorder compared with ∼25% in schizophrenia, suggesting that such composite scores would have limited diagnostic utility. Interestingly, parkinsonism at FEP (in unmedicated cohorts) was the motor feature most strongly associated with a schizophrenia diagnosis at follow-up.^89^

An important consideration, relevant to the practice of both psychiatrists and neurologists, is how improved understanding of motor manifestations in schizophrenia could assist in excluding or identifying neurological conditions in which psychotic and motor symptoms co-exist, such as Wilson’s disease, limbic encephalitis or Huntington’s disease. These cases are often challenging, given overlapping expertise in psychiatric and neurologic diagnosis is required. We highlight the need for more careful phenotyping of sensorimotor manifestations of schizophrenia to both avoid unnecessary investigations and avoid missed opportunities to detect and treat other neurological diseases. One area of particular interest would be better characterization of orofacial movements across psychotic conditions. Whilst dyskinetic movements in schizophrenia appear to have a predominance for the peri-oral region,^3,7^ it is not inconceivable that they might be confused with the orofacial dystonia (‘risus sardonicus’) seen in Wilson’s disease,^224^ the excessive tongue protrusion associated with neuroacanthocytosis syndromes^225^ or the complex plethora of orofacial motor disturbances observed in NMDAR antibody encephalitis,^226^ to name but a few examples. Here again, automated video assessment might make it feasible to perform detailed motor assessment at scale. Nadesalingam *et al*.^227^ have also demonstrated that the Positive and Negative Symptom Severity (PANSS) scale, commonly used in the assessment of patients with schizophrenia, can be adapted to form a motor-specific score comprising three of its components, namely mannerisms and posturing, motor retardation and disturbance of volition. Given psychiatric symptom rating tools may often overlook motor signs,^227^ a repurposed scale of this kind could be trialled as a means of detecting patients with significant motor features requiring further neurological assessment.

### Disease prognosis and monitoring

Sensorimotor examination might also play a role in prognostication. Schizophrenia is a condition with considerable variability in long-term outcomes.^18^ Increased burden of NSS at onset is associated with an elevated risk of having continuous symptoms thereafter,^15^ alongside reduced treatment response,^50,228^ poorer psychosocial functioning^228,229^ and increased service dependency at 10 years following onset.^228^ In a systematic review of 68 studies in patients across a range of disease stages (CHR, FEP, chronic schizophrenia), dyskinesia, parkinsonism and elevated NSSs were associated with psychiatric deterioration and poor functional outcome^230^ and the same three factors predicted poor prognosis at 21-year follow-up in a separate study of patients with FEP.^231^ In this respect, integrating sensorimotor examination into psychiatry assessments may also deserve exploration regarding their potential for early identification of those who may need early consideration of clozapine or novel therapies. Importantly, the association between higher rates of NSSs during FEP and non-remitting disease course has also been observed to be consistent across a range of final diagnoses,^15^ suggesting that sensorimotor phenotypes might have transdiagnostic value in respect to prognostication.

Monitoring of sensorimotor signs as a surrogate for psychopathology, is a context where remote assessment via wearable activity trackers holds much promise. In a meta-analysis of 38 studies using wrist-worn actigraphy in schizophrenia, lower motor activity and greater sleep duration correlated with negative symptoms whilst disorganized motor activity and sleep behaviour correlated with positive symptoms.^232^ Furthermore, digitally enabled footwear insoles have been demonstrated to detect abnormalities in stride length, cadence, speed and variability in patients with schizophrenia,^233^ and elsewhere similar gait abnormalities, measured utilizing a walkway embedded with motion sensors, have been shown to correlate with negative symptom severity and psychomotor slowing,^234^ parameters which in turn could be used as outcome markers. Similarly, degree of postural sway has been shown to correlate with overall symptom severity within a CHR cohort.^185^

### Prediction and monitoring of medication side effects

Whilst sensorimotor abnormalities at initial presentation might be expected to predict severity of motor side effects following initiation of medication, research in this area has yielded mixed results. Several studies specifically comparing rates of dyskinesia at FEP before and after treatment with antipsychotics have found no significant correlation,^10,65,219,235,236^ though follow-up was in general short (3 weeks to 6 months) so they may have failed to identify cases of TD which occurred later.^27^ Two studies have identified an association between spontaneous movement disorders and development of parkinsonism following medication initiation^10,237^; however, in one of these two, patients with more severe motor disturbance at presentation with FEP were also found to have worse prognosis in general,^10^ and other studies have demonstrated an improvement in motor features following antipsychotic treatment.^238,239^ Whilst more robust research is needed in this area, the lack of consistent findings may just highlight the degree to which motor dysfunction is a primary feature of schizophrenia rather than a side effect of antipsychotic treatment. Indeed, repeating rating scales for parkinsonism, dyskinesia, NSS and catatonia in 243 unmedicated patients during FEP and then at 21 years follow-up, Peralta *et al*.^89^ found that whilst rating in each domain at follow-up was significantly correlated to subsequent cumulative antipsychotic exposure, associations were no longer significant after adjusting for baseline sensorimotor dysfunction and illness severity.

Instrumental sensorimotor assessments do, however, show promise in the monitoring and quantification of motor side effects following D2R treatment. One validated method is digital characterization of handwriting kinematics, which can effectively quantify decrements in velocity scaling in Parkinson’s disease.^240^ PET studies also demonstrate significant correlations between decrements in handwriting area (micrographia) and proportion of D2R occupancy in patients with schizophrenia, compared before and after administration of haloperidol,^241^ risperidone^241,242^ and clozapine.^241^ Similar techniques effectively differentiate medicated from unmedicated patients,^219,240,243-245^ and predict D2R antagonist dosage in patients with schizophrenia,^244^ something that could not be determined through clinical examination alone.^244^ Wrist-worn actigraphy has also shown efficacy in identifying patients who develop akathisia acutely following antipsychotic treatment induction^246^ and again there are a host of digital smartphone-enabled technologies that might be similarly applied to detect motor medication side effects, including finger-tapping apps^247^ and physical activity monitors.^248^

### Treatment of sensorimotor symptoms

The possibility that sensorimotor dysfunction can be a primary (and disabling) manifestation of schizophrenia has implications for research into treatment. Interventions which benefit psychiatric symptoms should also be tested for their effect on sensorimotor features. As an example, there are mechanistic grounds to suggest that the muscarinic receptor partial agonist xanomeline-trospium (KarXT), which has recently shown efficacy in treating positive and negative psychotic symptoms,^249^ could also benefit motor symptoms, given that cholinergic interneurons are recognized to influence dopamine release in the striatum^250^ and modulation in muscarinic receptor function can ameliorate levodopa-induced dyskinesia in rodents.^251^ It also highlights the need for further research into active treatments for parkinsonian symptoms in schizophrenia, other than removal or switching of D2R antagonists. Levodopa (maximum dose of 600 mg/day) has been shown to improve parkinsonian symptoms in patients with evidence of nigrostriatal dysfunction on DaT neuroimaging.^115^ Whilst this treatment was not without risk of relapse, worsening of psychiatric symptoms only occurred in a minority of patients in this study [15.8% (3/19)],^115^ and future studies should focus on developing predictive tools to identify patients who are likely to respond positively to dopaminergic compounds without experiencing psychiatric deterioration. Another important area for further exploration is the role of clozapine in the management of motor disturbance in schizophrenia. While switching to clozapine appears to reduce TD severity^252^ and risk,^253^ it remains unclear whether this effect is solely due to its low D2R affinity or if clozapine directly prevents TD through other mechanisms. Elucidating the precise mechanism of clozapine’s action against TD could potentially lead to the development of novel therapeutic options for sensorimotor dysfunction.

Beyond pharmacological interventions, non-invasive TMS has emerged as a promising treatment modality for motor dysfunction in schizophrenia. Two recent randomized controlled trials demonstrated that repetitive inhibitory TMS of the supplementary motor area significantly improves psychomotor slowing in both schizophrenia^254^ and a transdiagnostic cohort,^255^ when compared with sham treatment. Evidence from case series also suggests that transcranial direct-current stimulation to the left DLPFC and temporoparietal junction may alleviate catatonia, though more robust research is needed to confirm efficacy.^256^

Revised understanding of sensorimotor dysfunction in schizophrenia also has implications for the application of functional neurosurgery. Deep brain stimulation (DBS) of the globus pallidus internus has demonstrated clinical utility in the management of treatment refractory TD and tardive dystonia.^257,258^ If dyskinesia and dystonia are not exclusively secondary to antipsychotic exposure, one could argue that DBS could mitigate ‘primary’ motor features of schizophrenia. Interestingly, beneficial effects of DBS on tardive dystonia can persist after cessation of stimulation,^259^ supporting the role of potentially reversible neuroplastic processes in motor disorders associated with schizophrenia.^110^ Furthermore, bilateral pallidal DBS stimulation in severe TD has also been found to improve hypokinetic motor symptoms,^257^ suggesting that parkinsonism in schizophrenia is another suitable target for DBS. Implanted electrodes in patients with tardive syndromes also offer opportunities to study the electrophysiological basis of the broad range of movement disorders and other symptoms observed in schizophrenia, for example to better understand whether alterations in beta oscillations^260^ or local field potentials^261^ relate to sensorimotor, cognitive or psychotic symptoms which could then be used as targets for therapeutic manipulation.

## Conclusions

In this review we have outlined the many ways in which the sensorimotor system is affected in schizophrenia. What emerges is a complex picture, where the phenomenology of motor disturbance is often confusingly characterized and direct causality typically unclear. The wide array of associated sensorimotor features seen, alongside their variability across the lifetime and between individuals with schizophrenia, also suggests that these sensorimotor phenomena are unlikely to be neatly explained by any one mechanistic hypothesis. The sensorimotor system appears to be a model through which the interaction between the many factors that underpin schizophrenia pathogenesis can be explored and, importantly, more objectively characterized using instrumental assessment. It is abundantly clear from clinical and experimental studies that all sensorimotor disturbances seen in schizophrenia cannot be simply explained by the acute and chronic effects of antipsychotic medication and that an improved understanding of sensorimotor features could have implications for the clinical management across the domains of risk assessment, diagnosis, prognostication, monitoring and treatment. It is hoped that the recent addition of a sixth sensorimotor systems category to the Research Domain Criteria by the National Institute of Mental Health will continue to foster further research in this area.^262^ As Halligan and David^263^ suggested with respect to the ‘cognitive neuropsychiatry’ of schizophrenia, the study of sensorimotor abnormalities would incentivize collaborative work between the fields of psychiatry, neurology and basic neuroscience. Indeed, schizophrenia could serve as a fertile ground for generating a broader ‘sensorimotor neuropsychiatry’ which might also have applications in the diagnosis and management of other conditions.
